# A decision aid for considering indomethacin prophylaxis vs. symptomatic treatment of PDA for extreme low birth weight infants

**DOI:** 10.1186/1471-2431-11-78

**Published:** 2011-09-03

**Authors:** Khalid M AlFaleh, Eman Al Luwaimi, Turki M AlKharfi, Saleh A Al-Alaiyan

**Affiliations:** 1 Department of Pediatrics (Division of Neonatology), College of Medicine, King Saud University, Riyadh, Saudi Arabia; 2 Department of Pediatrics (Division of Neonatology), King Faisal Specialist Hospital and Research Center, Riyadh, Saudi Arabia

**Keywords:** Indomethacin, patent ductus arteriosus, intraventricular hemorrhage, premature infants

## Abstract

**Background:**

Decision Aids (DA) are well established in various fields of medicine. It can improve the quality of decision-making and reduce decisional conflict. In neonatal care, and due to scientific equipoise, neonatologists caring for extreme low birth weight (ELBW) infants are in need to elicit parents' preferences with regard to the use of indomethacin therapy in ELBW infants. We aimed to develop a DA that elicits parents' preferences with regard to indomethacin therapy in ELBW infants.

**Methods:**

We developed a DA for the use of the indomethacin therapy in ELBW infants according to the Ottawa Decision Support Framework. The development process involved parents, neonatologists, DA developers and decision making experts. A pilot testing with healthy volunteers was conducted through an evaluation questionnaire, a knowledge scale, and a validated decisional conflict scale.

**Results:**

The DA is a computer-based interactive tool. In the first part, the DA provides information about patent ductus arteriosus (PDA) as a disease, the different treatment options, and the benefits and downsides of using indomethacin therapy in preterm infants. In the second part, it coaches the parent in the decision making process through clarifying values and preferences. Volunteers rated 10 out of 13 items of the DA positively and showed significant improvement on both the knowledge scale (p = 0.008) and the decisional conflict scale (p = 0.008).

**Conclusion:**

We have developed a computer based DA to assess parental preferences with regard to indomethacin therapy in preterm infants. Future research will involve measurement of parental preferences to guide and augment the clinical decisions in current neonatal practice.

## Background

### Burden of Illness

Due to its high rate of mortality and morbidity, prematurity is by far the most important issue in modern perinatal medicine. Patent ductus arteriosus (PDA) is very common among very low birth weight infants (VLBW), the delay in closure of the ductus is inversely related to gestational age varying from 20% in premature infants greater than 32 weeks up to 60% in extreme low birth weight infants (ELBW < 1000 g) [1,2]. PDA results in a significant left to right shunt with an increase in left ventricular output. Although it usually closes spontaneously by 5 days of age in most infants > 30 weeks' gestation, it remains patent by 5 days of age in more than two thirds of infants < 30 weeks [3].

Although controversial, observational data confirmed an association of PDA with the subsequent development of bronchopulmonary dysplasia (BPD) in premature infants [4,5]. It has also been demonstrated that large left to right shunting through the ductus results in a diastolic steal of blood flow to vital organs i.e. brain, kidneys, and intestines [6]. However, recent observational data failed to show an association of PDA with necrotizing enterocolitis (NEC) [7]. Failure of the ductal constriction has been shown to be associated with low superior venacaval flow (SVC) and subsequent occurrence of late intraventricular hemorrhage (IVH) [8]. Lastly, large left to right ductal shunting is associated with a significant increase in pulmonary blood flow and serious pulmonary hemorrhage [9,10].

#### Indomethacin prophylaxis vs. treatment for symptomatic PDA; the clinical dilemma

There is now a substantial body of literature available to evaluate the role of indomethacin prophylaxis in the management of the premature infant. Indomethacin prophylaxis reduces the incidence of symptomatic PDA by 55%, severe grade III and IV IVH by 35%, and the need for surgical PDA ligation by 50% [11,12]. In addition a recent ancillary analysis on the database of the trial of indomethacin prophylaxis in the preterm infants (TIPP trial), showed a significant reduction of serious clinically significant pulmonary hemorrhage during the first week of life [13].

However, this significant reduction in symptomatic PDA, severe IVH, and serious pulmonary hemorrhage did not translate in reduction of mortality, BPD, and more importantly the rates of long term neurosensory outcome.

As a result of the data presented above, clinicians caring for premature infants are left uncertain of what would be the most sensible approach to the use of indomethacin prophylaxis in the preterm. Some clinicians would justify its use based on the reduction of important intermediate morbidities. On the other hand, others would refrain employing a prophylactic strategy bearing in mind the lack of long term improvement in mortality rate and neurosensory outcome [14].

Patient decision aids can improve the quality of decision-making, reduce decisional conflict and help patients become involved in decision making by providing information about the options and outcomes and by clarifying personal values. They are designed to complement, rather than replace, counseling from a health practitioner. The medical literature has observed an increasing number and utilization of decision aids in patients management in various fields of medicine [15-17].

### Objective

To develop a decision aid to elicit parents of premature infants' preferences with regard to indomethacin prophylaxis vs. symptomatic treatment in the management of their infants.

## Methods

### Structural development

The framework is an evidence-based, practical, midrange theory for guiding parents making health or social decisions. It follows the Ottawa Decision Support Framework, and is based on the Ottawa Personal Decision Guide (OPDG) [18]. The DA supports decision making through providing information about the disorder, its management and its outcomes through a computer based interface.

### Evidence Sources

Information about the outcomes of indomethacin prophylaxis in ELBW infants were driven from the best evidence reported in the published meta-analysis of indomethacin prophylaxis use in preterm infants. More than 19 randomized trials were included in the report [11,12].

### Platform design

A web-based DA format was chosen to allow for broader utilization, easier access. A professional web designing establishment developed the platform of the DA using PHP scripting language and MySql database and made available on the World Wide Web.

### Experts' feedback

Feedback of the DA structure and content was sought of neonatologists, DA experts commenting on the structure and content of the DA. Eight experts were approached by e-mail (5 international and 3 local). Experts provided constructive feedback with some modification regarding the scientific data presented, language and general layout of the DA.

### Parents' feedback

We conducted interviews with 10 expecting mothers (ten mothers of infants in our NICU were randomly selected to participate in this pilot study). Each participant answered specific questions and provided general comments. An example of a general comment; " it was a simple and an interesting way to understand the condition of my child, it is nice to see such interactive tools to cover our health concerns about our children". Modifications were made based on the participant feedback. Modifications were mainly related to the DA language, numeric data presentation, and other scientific information.

### Pilot testing of the Decision Aid

Ten mothers (other than those involved in the parents feedback) used the DA as if they were to make a real life decision about the use of indomethacin therapy. Initially, they were introduced to the concept of prematurity and PDA followed by explaining the role of indomethacin therapy as a management option. These participants then completed 3 instruments: (1) an evaluation questionnaire; (2) a knowledge scale before and after use; and (3) a validated decisional conflict scale (before and after use) [18]. The evaluation questionnaire addressed 9 features of the DA that participants rated on 5-point Likert scale (1 for highest value, 3 for neutral value and 5 for lowest value). The knowledge scale consisted of 8 questions we developed specifically about prematurity, PDA, the required time and the need for assistance in using the DA.

### Statistical analysis

Tests utilized in this study were a one sample *t*-test for the 5-point Likert scale and paired *t*-test to compare the pre and post means for the knowledge and decisional conflict scales. A two sided *P *value < 0.05 was considered statistically significant.

The study was approved by the ethics committee, King Saud University, college of medicine, Riyadh, Saudi Arabia.

## Results

### The Decision Aid (DA)

#### Structure

DA was structured in two parts; the first part provides medical information about: the use of the DA, prematurity and its complications, PDA, and the use of indomethacin therapy in ELBW infants (prophylactic indomethacin and symptomatic PDA treatment), chance of intermediate outcomes (risk of PDA, surgical PDA ligation, severe IVH), chance of long term outcomes (survival, neurosensory impairment) for both options, and potential adverse effects (gastrointestinal perforation, renal impairment) associated with indomethacin prophylaxis use, all presented in a systematic and balanced way through a series of screen shots, that included several images and simplified language in both paragraphs and bullet points.

In the second part; the DA coaches the parents in the decision making process regarding the use of indomethacin for their preterm infants through: case scenarios of the PDA and side effects of indomethacin, clarification of the patient's own values for each benefit and harm; and assistance in the final decision making, a verbal description of uncertainty was utilized (e.g. likely) rather than a numeric one (e.g. 0.80) in order not to overload participants with numbers and because evidence shows that verbal is as effective as numeric communication. However, numeric values were displayed on the decision aid instrument [17].

#### Rating values

The parents will assign the value of each potential outcome associated with indomethacin therapy. The DA presents the values as a horizontal feeling-thermometer with values ranging from 0 (worst outcome imaginable i.e. death) to 100 (best health condition imaginable) by increments of 1 unit. The parents move the cursor of the scale to assign the value for a specific outcome. Their assigned value will show in the box adjacent to the scale.

#### Decision making models

Later, the DA allows the parents to choose one of the two possible decision making models. After integrating the information provided, their values the parents will provide a choice '' to use '' or '' not to use'' indomethacin as prophylaxis for their ELBW infant as an individual choice. The parents have the choice to leave the decision aid at any stage and leave the decision to their health care provider.

#### Navigation

User friendly navigation was utilized. The parent can access the first page of any section from any page of the DA, and all pages of a particular section from any page of that section.

#### Pilot testing of the Decision Aid

Characteristics of the mothers who participated in the pilot testing of the DA are listed in Table 1.

**Table 1:** Baseline characteristics of pilot testing mothers

Variable		Respondents (N = 10)
		**Mean**
**Age mean (SD)**		36.2 (7.76)
**Educational level N (%)**		
Less than high school		1(10)
High school		1 (10)
College		2 (10)
Graduate degree		6 (60)
		
**Computer user**		8 (80)
**Internet user**		8 (80)

The results of the pilot testing are listed in Table 2. Positive feedback regarding the design, pictures, understandability, user friendliness, perceived required time, explanations, and amount of information in the DA was provided by the participants. They felt comfortable and satisfied while using it. Both the knowledge and the decisional conflict scales improved significantly after review of the DA. The mean time needed to finish reviewing the DA was 19 minutes, only one of the volunteers needed assistance in using the appropriate buttons to navigate the DA.

**Table 2:** Results of pilot testing

Variable	Value	P value
Design	1.2 (0.422)	P < 0.0001
User friendliness	1.1 (0.316)	P < 0.0001
Pictures	1.3 (0.675)	P < 0.0001
Understandability	1.0 (0.0)	P < 0.0001
Amount of information	1.2 (0.422)	P < 0.0001
Knowledge improvement	1.0 (0.0)	P < 0.0001
Perceived required time	1.4 (0.516)	P < 0.0001
Decision Aid helpfulness	1.3 (0.675)	P < 0.0001
Satisfaction	1.4 (0.699)	P < 0.0001
		
Knowledge scale pre*	1.7 (1.636)	P < 0.0001
Knowledge scale post	7.6 (0.699)	
		
Decisional Conflict scale pre **	25.4 (7.545)	P < 0.0001
Decisional Conflict scale post	0.6 (1.349)	
		
Number of minutes to complete the DA mean (SD)	19 (5.164)	N/A
Need for assistance to use the DA n (%)	4 (40%)	
*knowledge scale total score is 10		
** Decisional conflict scale total is 40		

## Discussion

We have developed a computer based interactive DA for considering indomethacin therapy in the management of ELBW infants following a detailed well structured methodology based on the Ottawa Decision Support Framework. The DA will help parents of ELBW infants have an informed decision without the presence of their health care provider with regard to whether to offer or decline indomethacin prophylaxis in the management of their infants. The DA will also augment further research in evaluating parents preferences with regard to indomethacin therapy based on a reliable tool which will then completes the three circles (experience, research evidence, and patient preferences) of evidence based medicine in the area of indomethacin prophylaxis in preterm infants. Parents evaluated our DA rated most of its features positively and showed a significant improvement in their knowledge, decisional conflict scales.

Our DA is the first to our knowledge in neonatal care; it followed a rigorous well defined developmental process, and offers the ease, accessibility and convenience to users since it is web based. It can be utilized in the care of preterm infants starting at the antenatal visit. Our DA however has some limitations; first, our pilot testing involved ten subjects that makes it difficult to generalize the results for a wide spread use. Second, the computer, web based design although advantageous to many parents in the modern age makes it difficult to be utilized by non computer users. Third, a limitations of the DA development process is the lack of real life decision making of a parent of a preterm infant admitted in the intensive care unit, our future research will involve eliciting decision preferences of indomethacin therapy including pregnant women, women at risk of preterm delivery and parent of admitted extreme low birth weight infants in neonatal intensive care unit to test the impact of real life situation in the decision making process.

These findings will be taken as a step for designing a future project that involves evaluating parental preferences with regard to indomethacin therapy in ELBW infants in a large representative sample to help guide the practice of neonatal practitioners.

## Conclusion

We have developed a computer based DA to assess parental preferences with regard to indomethacin therapy in preterm infants. Future research will involve measurement of parental preferences to guide and augment the clinical decisions in current neonatal practice.

## Competing interests

The authors declare that they have no competing interests.

## Authors' contributions

KA and EA conceived, designed and collected the data. All authors (KA, EA, TA, SA) contributed to the analysis, interpretations of the data, drafting and revision of the manuscript. All authors have read and responded to the referees' comments. All authors approved the final manuscript.

## Funding source

This project was supported by a shared grant of the center of medical research and the neonatal research chair at the college of medicine, King Saud University, Riyadh, Saudi Arabia.

## Pre-publication history

The pre-publication history for this paper can be accessed here:

http://www.biomedcentral.com/1471-2431/11/78/prepub
